# Investigating the potential of augmented reality game-based instruction on Chinese EFL learners’ vocabulary learning and academic enjoyment

**DOI:** 10.3389/fpsyg.2026.1861679

**Published:** 2026-08-10

**Authors:** Huan Wang, Guofen Zhu

**Affiliations:** 1 School of Foreign Languages, Jinling Institute of Technology, Nanjing, China; 2 School of Marxism, Nanjing University of Science and Technology, Nanjing, China

**Keywords:** academic enjoyment, augmented reality, game-based learning, mixed-methods, vocabulary learning

## Abstract

With the growing interest in integrating technology into language teaching, Augmented Reality (AR) has emerged as a promising tool for enhancing learning experiences. However, there is limited research examining its impact on Chinese English-as-a-Foreign-Language (EFL) learners’ vocabulary learning and academic enjoyment. The study aims to explore how innovative teaching approaches, such as AR game-based learning, can enhance Chinese EFL learners’ vocabulary acquisition and overall academic enjoyment. Two intact classes of 80 learners each from a large university in China were randomly assigned to either an experimental group, which received AR game-based instruction, or a control group, which received traditional teacher-fronted instruction. A concurrent mixed-methods design was employed. Quantitative data were collected through pretest and posttest vocabulary measures and analyzed using independent-samples t-tests. Findings indicated that although the two groups were comparable at the pretest stage, the experimental group significantly outperformed the control group on the posttest, indicating AR game-based instruction’s effect on vocabulary learning. Qualitative data were gathered through learners’ narratives and semi-structured interviews and analyzed thematically to explore their perceptions and experiences of academic enjoyment in the AR-supported learning environment. The analysis revealed three major themes: perceived heightened interest through novelty and immersion, perceived enhanced satisfaction through clearer understanding and vocabulary retention, and perceived increased motivation through interactivity and game-like engagement. Taken together, the findings suggest that AR game-based instruction can support both vocabulary development and positive emotional engagement in EFL learning. The study offers implications for teachers, curriculum developers, and educational decision-makers seeking to integrate innovative and emotionally supportive technologies into language education.

## Introduction

In an era marked by growing global mobility and increasing patterns of migration, proficiency in more than one language has become an important asset. Among world languages, English has assumed a particularly prominent role as a medium of international communication, academic participation, and professional development across a wide range of social and cultural settings (Christou et al., 2024). Nevertheless, traditional forms of language teaching often prove insufficient for maintaining learners’ interest throughout the learning process. Against this backdrop, innovative pedagogical practices—especially the use of games—have attracted increasing interest for their potential to make language learning more engaging, interactive, and immersive (Hung et al., 2018).

The increasing presence of digital technologies in socio-educational contexts has significantly reshaped both instruction and learning (Dai and Liu, 2024). Among recent developments in computer-assisted language learning, AR has been described as a key technological link for twenty-first-century education (Kroeker, 2010) and is widely regarded as offering substantial pedagogical value (e.g., Altinpulluk, 2019; Fidan and Tuncel, 2019; Ndrurua et al., 2025). Unlike virtual reality (VR), which places users inside a fully simulated environment (Bower et al., 2014), AR overlays digital elements onto the real world, thereby enriching learners’ sensory engagement and supporting a more effective learning environment (Specht et al., 2011). Extending this discussion, AR facilitates a more immersive form of human–machine interaction by enabling users to encounter virtual elements through mobile devices and computer-based interfaces, often via cameras and embedded imaging systems. By drawing on high-bandwidth internet, GPS, touchscreens, and sensor-based technologies, AR integrates display, interactivity, and instructional support within a single mobile platform. In doing so, it creates smart learning environments that are context-sensitive and responsive to sensory input, while also affording flexible opportunities for interaction and collaboration. These affordances have transformed teaching and learning practices by enabling learners to engage with enriched forms of input and apply them in multisensory, authentic-like contexts (Saleh et al., 2025).

Research has shown that AR can positively influence learners’ motivation and attitudes (Norouzifard et al., 2022), academic performance (Lee, 2012), English learning (Ndrurua et al., 2025), and teacher education programs (Salimi and Ranjbar, 2024). However, these findings have not always been conclusive. For instance, using a quasi-experimental approach, Ou Yang et al. (2023) investigated the comparative effectiveness of AR Bot and Scratch in programming learning. The study included 41 first-year university students in the experimental condition, who used AR Bot, and 34 first-year university students in the control condition, who learned with Scratch. The researchers compared the two tools in terms of students’ internal learning processes, especially enjoyment of learning, their computational thinking (CT) competencies, and their academic achievement. The results demonstrated that students in the AR Bot condition reported greater learning enjoyment and performed better in algorithm design and algorithm efficiency than those in the Scratch condition. Nevertheless, no significant differences emerged between the two groups with respect to problem decomposition or academic achievement. The study also revealed that enjoyment of learning was positively associated with CT skills, though not with academic achievement. Moreover, among the CT subskills, problem decomposition and algorithm design significantly predicted academic achievement, whereas algorithm efficiency did not. In another study, Raghav Bhat et al. (2025) argued that while AR enhanced engagement through enriched input and appealing visual design, it did not produce corresponding gains in learning outcomes, leading the authors to call for more pedagogically effective implementation. This contrast implies that AR’s effectiveness in education is not an inherent property of the technology itself, but rather a function of how deliberately and appropriately it is designed for instructional purposes.

As noted above, the use of games as innovative educational practices has entered into prominence in recent years as they have the potential to make the teaching-learning process more engaging, interactive, and immersive (Hung et al., 2018). Within language education, digital game-based language learning (DGBLL) has emerged as a valuable approach for enhancing linguistic development. Previous research has linked DGBLL to a range of positive outcomes, including greater motivation to learn, improved reading comprehension, and enhanced short-term memory (Xiong et al., 2024; Zou et al., 2021). When effectively scaffolded by teachers, such environments can enable learners to develop language knowledge and skills through context-aware, multimodal, and highly interactive learning experiences (Hung et al., 2018). Xu et al. (2020) in a recent review, reported that a large share of DGBLL research has been situated in the United States and Taiwanese educational contexts, whereas many other learning settings remain underexplored.

In recent decades, mobile games have emerged as a prominent educational technology and an important area of inquiry in language learning research (Chen et al., 2019; Wu, 2021). Mobile game-based language learning (MGBLL) is often viewed as a promising approach because it brings together the accessibility and ubiquity of mobile devices with the immersive and engaging nature of digital games (Elaish et al., 2019; Godwin-Jones, 2014). Empirical research has indicated that mobile games (see Kazu and Kuvvetli, 2024; Li, 2021; Roohani and Heidari Vincheh, 2021), can contribute to the development of various language skills, such as speaking, reading, writing, vocabulary, grammar, pronunciation, and literacy (e.g., Amorim et al., 2022; van Uittert et al., 2022).

In total, the reviewed literature showcases that AR and DGBLL enhance various facets of language learning. Despite these contributions of AR, DGBLL and MGBLL to educational growth, the extent to which AR game-based learning enhances vocabulary learning particularly within Chinese context is not understood thoroughly. Hung and Yeh (2023) provides one of the few investigations in this area. These EFL researchers found that learners who participated in AR game-supported instruction outperformed those in paper-and-pen-based instruction on tests of vocabulary knowledge and creative thinking. Similarly, Jia et al. (2025) demonstrated the advantages of AR-supported game-based interventions for the learning of academic English vocabulary. Drawing on delayed posttest data, they showed that the AR-supported game-based model was particularly effective in promoting long-term vocabulary retention. In a related study, Khazaie and Ebadi (2025) examined the role of an AR-supported gamified flipped learning context in reading comprehension and reported that this instructional model significantly improved learners’ understanding of medical English texts. Likewise, Namaziandost and Çakmak (2026) investigated whether an AR game-supported flipped learning model could shape important aspects of language learning, including cognitive load, vocabulary retention, and oral fluency. The results indicated that learners in the experimental group experienced lower cognitive burden and demonstrated superior vocabulary retention and speaking fluency compared with those in the control group.

Recently, scholars in second language acquisition (SLA) have shown growing interest in the role of positive emotions in language learning, particularly enjoyment. Enjoyment is increasingly conceptualized as a multifaceted emotion that reflects individuals’ sustained drive to succeed despite the presence of challenging tasks (Dewaele and MacIntyre, 2016). More specifically, it denotes the positive feelings of pleasure and happiness experienced while engaging in an activity (Tahmouresi and Papi, 2021). In academic settings, enjoyment is typically defined as students’ satisfaction with their educational environment. It represents a positive emotional disposition toward the overall institutional learning context and constitutes an important dimension of school-related well-being (Hascher and Hagenauer, 2011). Moreover, academic enjoyment is closely connected to learning activities, course content, and interpersonal relationships with teachers, classmates, and peers (Lumby, 2011; Ömeroğulları and Gläser-Zikuda, 2021).

Academic enjoyment can be understood as the emotional response associated with the successful accomplishment of a goal or the completion of a task (Pekrun, 2006). It is increasingly viewed as a multifaceted construct comprising psychological, cognitive, motivational, interpersonal, and bodily dimensions (Elahi Shirvan et al., 2020). These emotional experiences can influence learners’ motivation, their interactions with teachers and peers, and their physical well-being (Dewaele and MacIntyre, 2016; Jiang and Dewaele, 2019). Importantly, enjoyment is not static; rather, it is subject to change over time. Dewaele et al. (2018) noted that language-related academic enjoyment develops dynamically as a function of both individual learner characteristics and the surrounding educational environment. Research has also underscored the role of teacher–student relationships in fostering academic enjoyment in classroom settings (e.g., Dewaele et al., 2018; MacIntyre et al., 2019). This interpretation is reinforced by Khajavy et al. (2018), who found that learners with higher levels of enjoyment tend to participate more actively and engage more frequently with peers.

This study is theoretically grounded in two complementary perspectives: constructivism and the Affective Filter Hypothesis. Together, these frameworks provide a strong foundation for explaining how augmented reality game-based instruction may enhance Chinese EFL learners’ vocabulary development and academic enjoyment. From a constructivist perspective, learning is understood as an active process in which learners build knowledge through engagement, interaction, and meaning-making rather than through passive reception of information. In the context of AR, this view is especially relevant because AR-based learning environments allow students to interact dynamically with instructional content and connect new knowledge to their prior experiences. As Dunleavy and Dede (2014) note, learning is shaped by the particular contexts in which it occurs, including the relationships among individuals, environments, objects, actions, and cultural elements. In this sense, AR does not merely deliver content; it situates learning within meaningful contexts that can make abstract concepts more concrete and accessible. In addition, constructivist learning emphasizes authentic activity, learner participation, and interaction, while situated learning highlights the importance of knowledge construction within socially and contextually grounded settings. AR supports these principles by creating immersive and context-sensitive learning experiences that encourage learners to explore, experiment, and discover knowledge actively. As argued by Robinson and Coltz (2013), AR can promote constructivist learning by enabling learners to engage directly with interactive materials. Similarly, Wang et al. (2018) suggest that such environments help learners combine unfamiliar concepts with their existing knowledge structures, thereby promoting individualized and self-directed learning. In line with this, Belda-Medina (2022) emphasizes that AR can foster learner-centered and experiential educational experiences that contribute to deeper understanding. Therefore, constructivism provides an appropriate lens for explaining how AR game-based instruction may support vocabulary learning through active participation, contextualized practice, and meaningful engagement.

The study is also informed by Krashen’s (1982) Affective Filter Hypothesis, which offers an important explanation for why learners may succeed or struggle in second or foreign language acquisition. Accordingly, emotional conditions play a crucial role in determining how effectively learners process and acquire language input. Krashen (1982) proposes that learners possess an “affective filter” that can either facilitate or hinder language learning depending on their emotional state. When learners experience negative feelings such as anxiety, fear, boredom, embarrassment, shame, or low self-confidence, this filter becomes stronger, which reduces their ability to absorb and internalize linguistic input. By contrast, when learners feel relaxed, motivated, joyful, confident, proud, or enthusiastic, the affective filter is lowered, allowing language input to be received and processed more effectively. Within this framework, enjoyment becomes especially significant because it creates favorable emotional conditions for language acquisition. In other words, when students experience academic enjoyment during classroom activities, they are more likely to engage openly with the target language and benefit more fully from instructional input. For this reason, the Affective Filter Hypothesis is particularly relevant to the present study, as AR game-based instruction may create an enjoyable, low-stress, and motivating learning environment that not only supports vocabulary development but also enhances learners’ academic enjoyment.

Taken together, constructivism and the Affective Filter Hypothesis provide complementary lenses for understanding the potential role of augmented reality game-based instruction in EFL vocabulary learning. Constructivism explains the cognitive and instructional pathway through which learning may occur. From this perspective, AR game-based instruction can support vocabulary development by allowing learners to interact actively with multimodal content, connect new lexical items with visual and auditory representations, engage in contextualized practice, and construct meaning through exploration rather than passive reception. In this way, vocabulary learning becomes an active, situated, and learner-centered process.

The Affective Filter Hypothesis complements this explanation by accounting for the emotional conditions that may facilitate or hinder this learning process. Even when input is available, learners may not process it effectively if they experience anxiety, boredom, low confidence, or lack of motivation. AR game-based instruction may help lower such affective barriers by creating a more enjoyable, motivating, and less monotonous learning environment. Features such as interactivity, immediate feedback, game-like challenge, and immersive visualization can increase learners’ interest and confidence, thereby making them more receptive to language input.

Therefore, the two theories are not treated as separate or competing explanations in this study. Rather, they address two interrelated dimensions of the same instructional process. Constructivism explains how learners can actively build vocabulary knowledge through interaction with AR-supported materials, while the Affective Filter Hypothesis explains why learners may become more emotionally ready to engage with and internalize that input. Their complementarity is particularly relevant to the present study because the research examines both a cognitive outcome, vocabulary learning, and an affective outcome, academic enjoyment. The theoretical assumption is that AR game-based instruction may be effective when it combines meaningful knowledge construction with positive emotional engagement.

Based on the complementary insights of constructivism and the Affective Filter Hypothesis, the present study is guided by a conceptual framework that links augmented reality game-based instruction with technology adoption, self-directed learning, motivation, and vocabulary learning outcomes. In this framework, technology adoption refers to learners’ willingness to engage with and use AR-supported tools as part of the instructional process. Because AR game-based learning environments are interactive, visually enriched, and feedback-oriented, they may increase learners’ readiness to participate in technology-mediated learning. At the same time, such environments may promote self-directed learning by encouraging learners to explore vocabulary items actively, repeat tasks, monitor their own performance, and respond to immediate feedback with less dependence on continuous teacher explanation.

The framework further assumes that technology adoption and self-directed learning are closely related to learners’ motivation and academic enjoyment. When learners are willing to use the technology and are able to engage actively with learning tasks, they may experience greater interest, satisfaction, and involvement in the learning process. In turn, these positive affective experiences may support stronger engagement with vocabulary learning and contribute to improved learning outcomes. The framework is intended as a theoretical guide for understanding the interrelationship among these constructs in the present study rather than as a fully tested causal model. It helps explain how AR game-based instruction may function not only as a technological tool, but also as a pedagogically structured environment that supports active learning, emotional engagement, and vocabulary development.

Despite growing interest in augmented reality and game-based approaches in language education, there remains insufficient empirical evidence regarding how augmented reality game-based instruction can support Chinese EFL learners’ vocabulary learning and academic enjoyment in a pedagogically meaningful way. Existing research has generally shown that AR can increase engagement, motivation, and, in some cases, learning outcomes; however, the findings are still mixed, and relatively few studies have examined the combined cognitive and affective impact of AR game-based instruction in the Chinese EFL context, particularly with respect to vocabulary development and enjoyment in academic settings. This gap is important because vocabulary knowledge is central to successful language learning, while academic enjoyment plays a key role in sustaining learners’ participation, motivation, and openness to language input. Accordingly, the present study seeks to address this problem by investigating the potential of augmented reality game-based instruction to enhance Chinese EFL learners’ vocabulary learning and academic enjoyment, while also contributing context-sensitive evidence to the literature on technology-enhanced language learning and offering pedagogically grounded insights into how AR-based instructional design may foster both improved learning outcomes and more positive emotional experiences in the EFL classroom. Therefore, this study addresses these research questions:

1. Does augmented reality game-based instruction affect Chinese EFL learners’ vocabulary learning?
2. How do Chinese EFL learners perceive and experience academic enjoyment in augmented reality game-based instruction?

The two research questions were developed directly from the theoretical foundations of the study. The first research question, which examines the effect of augmented reality game-based instruction on vocabulary learning, is informed by constructivism. From this perspective, vocabulary learning is expected to improve when learners actively construct meaning through interaction with multimodal, contextualized, and learner-centered materials. The AR game-based environment was therefore designed to provide learners with opportunities to connect lexical form, meaning, pronunciation, visual representation, and contextual use through active engagement. The second research question, which explores learners’ academic enjoyment, is informed by the Affective Filter Hypothesis. This perspective suggests that positive emotions may make learners more receptive to language input, whereas negative emotions may restrict engagement and learning. Accordingly, the study investigated not only whether AR game-based instruction improved vocabulary learning, but also how learners experienced enjoyment within this instructional environment.

We believe that the present study is timely and important because it addresses an underexplored area at the intersection of augmented reality, game-based language learning, and Chinese EFL education by examining both a cognitive outcome, namely vocabulary learning, and an affective outcome, namely academic enjoyment. In doing so, it extends current scholarship beyond the predominant focus on achievement alone and responds to the need for more pedagogically grounded evidence regarding how AR game-based instruction functions in actual language learning contexts. By investigating whether this instructional approach supports vocabulary development and how learners perceive and experience enjoyment within it, the study is expected to contribute a more holistic understanding of technology-enhanced language learning that acknowledges both performance and emotional engagement as central to successful language acquisition. The potential implications of the study could therefore be both theoretical and practical: theoretically, the findings may enrich ongoing discussions of constructivist learning and affective dimensions of second language acquisition in digitally mediated environments; practically, they may offer useful guidance for EFL teachers, curriculum designers, material developers, and educational decision-makers seeking to integrate AR-based game instruction in ways that are not only innovative but also educationally meaningful, learner-centered, and emotionally supportive.

## Methods

### Design

This study employed a concurrent mixed-methods design to provide a more comprehensive understanding of the effects of augmented reality game-based instruction. Quantitative data were collected to examine whether this instructional approach had a significant effect on Chinese EFL learners’ vocabulary learning, while qualitative data were gathered to explore learners’ academic enjoyment within the AR game-based learning environment. By collecting both types of data concurrently, the researchers tried to investigate the cognitive and affective dimensions of the instructional intervention in an integrated manner.

This design was selected because the study examined both the effect of AR game-based instruction on vocabulary learning and learners’ experiences of academic enjoyment. The quantitative strand was appropriate for addressing the first research question because it allowed comparison of vocabulary performance between the experimental and control groups before and after the intervention. The qualitative strand was appropriate for addressing the second research question because academic enjoyment is an affective and experiential construct that requires learners’ own accounts to be understood in depth.

### Context and participants

The study was conducted at a university in China. Participants were recruited from two intact undergraduate EFL classes. Initially, each class included 82 learners, resulting in a total of 164 students. However, two learners in each condition were absent during the testing sessions and were therefore excluded from the final analysis. As a result, the final sample consisted of 160 students, with 80 learners in the experimental group and 80 learners in the control group. The use of intact classes was adopted to avoid disrupting the normal instructional organization of the university. The two intact classes were randomly assigned at the class level to one of the two instructional conditions. One class was assigned to the experimental condition and received augmented reality game-based vocabulary instruction, whereas the other class was assigned to the control condition and received traditional teacher-fronted vocabulary instruction. Thus, randomization was conducted at the class level rather than at the individual learner level. Additionally, the sample size was considered adequate for the purpose of the study. Because the research was conducted in a natural university classroom setting, the sample was based on two intact EFL classes rather than on individual-level recruitment from the wider student population. The balanced group size was appropriate for the statistical comparisons between the two instructional conditions.

All participants were native speakers of Chinese and had neither traveled to nor resided in an English-speaking country for longer than two weeks, thereby reducing the possible influence of extended immersion in English-speaking settings. Participants were between 18 and 24 years old. To ensure relative homogeneity in initial language proficiency, all learners took the Quick Placement Test (Oxford University Press and University of Cambridge Local Examinations Syndicate, 2001), and the results indicated that all of them were at the A2 level. This relative comparability in both age and proficiency provided an appropriate foundation for examining the effects of the instructional intervention on vocabulary learning and academic enjoyment.

In addition, this study was conducted in accordance with the Declaration of Helsinki. The study was reviewed and approved by the School of Foreign Languages, Jinling Institute of Technology. Prior to participation, informed consent was obtained from the participants. The learners were informed about the voluntary nature of participation, and the confidentiality of their responses. Participants were also assured that the data would be used only for research purposes and that their identities would not be disclosed in any part of the study.

### Instruments

Several instruments were employed to address the quantitative and qualitative research questions. First, all participants completed the Quick Placement Test to establish relative homogeneity in English proficiency before the intervention. For the instructional treatment, the experimental group used Vocabulary Builder AR, a pre-existing third-party mobile application developed by 360ed. The application was installed on participants’ Android or iOS devices and used in its unmodified form.

The research team did not develop, program, customize, or modify Vocabulary Builder AR. The researchers did not control the application’s source code, image-recognition database, three-dimensional models, animations, audio files, user interface, interaction commands, feedback, scoring, or other game functions. Their role was limited to selecting the target vocabulary items and corresponding designated image cards, organizing their instructional sequence, and integrating the application’s existing features into the 14-session teaching procedure. Participants interacted only with Vocabulary Builder AR and its designated physical image cards.

Each designated card contained a printed image (also available online) associated with a target vocabulary item. During the instructional activities, learners opened the AR mode in Vocabulary Builder AR and positioned the device camera over the corresponding card. When the application recognized the scanned image, the preloaded digital model appeared on the device screen. Learners could view and interact with the model where the application permitted, listen to the word’s pronunciation, and engage with the application’s built-in activities. The image cards therefore served as visual image-recognition targets. Following the AR presentation, learners completed the application’s existing game-based tasks, including recognition, matching, spelling, and selection activities, and received the feedback and scores generated by the application.

To measure vocabulary learning, the researchers developed an 80-item vocabulary test consisting of four sections: blank-fill items, matching items, multiple-choice items, and short-answer items, with 20 items in each section. Each correct answer was awarded 0.25 points, yielding a total possible score ranging from 0 to 20. Three experienced English teachers, each of whom had at least 10 years of teaching experience and held at least a masters’ degree in TESOL and/or TEFL verified the test’s content validity. The test’s reliability was estimated through the KR-21 formula because all items were scored dichotomously as correct or incorrect. The resulting reliability coefficient was 0.86, indicating a high level of internal consistency. In addition, a parallel form of the test with the same number and type of items was constructed to assess learning gains. Its content validity was established through the same expert-review procedure, and its reliability was also found to be satisfactory (*r* = 0.83).

During the qualitative stage, learner narratives and semi-structured interviews were used to explore participants’ academic enjoyment in the AR game-based learning environment. The narrative task invited learners in the experimental group to provide written reflections on their emotional and learning experiences during the intervention. The narrative prompts addressed issues such as: how they felt while learning vocabulary through the AR game-based activities, which aspects of the AR experience they found enjoyable or unenjoyable, whether the activities increased their interest and participation in English learning, and how the AR-based tasks differed from their previous vocabulary learning experiences. To further deepen this exploration, semi-structured interviews were conducted with a subset of learners. The interview protocol included open-ended questions such as: “How did you feel when using the AR game-based application to learn vocabulary?” “Which features of the AR activities did you enjoy the most, and why?” “Did the AR-supported lessons make vocabulary learning more interesting or motivating for you?” “Were there any parts of the experience that reduced your enjoyment or made learning difficult?” and “In what ways was this experience different from traditional teacher-fronted vocabulary instruction?” These questions were designed to elicit detailed accounts of learners’ emotional engagement and perceptions of the instructional experience. It is of utmost importance to acknowledge here that to ensure that the participants’ limited English proficiency did not constrain the quality or depth of the qualitative data, they were allowed to respond to both the narrative prompts and the semi-structured interview questions in Chinese. This decision was made to enable the learners to express their thoughts, feelings, and experiences more freely and accurately, particularly when reflecting on their academic enjoyment in the augmented reality game-based instructional context. After data collection, the Chinese excerpts were translated into English for reporting purposes. To enhance the accuracy and trustworthiness of the translated data, the English versions were carefully checked by experienced English teachers, the same experts who had previously examined the vocabulary tests.

Several procedures were used to enhance the trustworthiness of the qualitative data and thematic analysis. Credibility was supported through the use of two qualitative data sources: learner narratives and semi-structured interviews. The use of both sources allowed the researchers to compare recurring patterns across written reflections and interview accounts. In addition, the narrative prompts and interview questions were reviewed by specialists in language education to ensure their relevance, clarity, and alignment with the second research question. The interview guide was also piloted with a small group of learners similar to the target participants, and unclear wording was revised before the main data collection. Member checking was used by sharing summaries of the interview content with participants so that they could confirm whether the researchers’ interpretations accurately reflected their views.

Dependability was addressed by following a consistent data collection and analysis procedure. All interviewees were asked comparable core questions, while follow-up questions were used when clarification or elaboration was needed. The researchers maintained detailed records of the narrative prompts, interview protocol, audio recordings, transcriptions, translations, coding decisions, and theme development. The narratives and interview transcripts were coded independently by two researchers. Inter-coder agreement was high, with a Cohen’s kappa coefficient of 0.85, indicating strong agreement between the coders. Any disagreements were discussed until consensus was reached.

Confirmability was strengthened through the maintenance of an audit trail documenting the movement from raw data to initial codes, categories, and final themes. This procedure helped ensure that the themes were grounded in participants’ accounts rather than in the researchers’ assumptions. To further reduce interpretive bias, the researchers repeatedly returned to the original narratives and interview transcripts when refining the themes. Transferability was supported by providing detailed information about the study context, participants, instructional treatment, qualitative data sources, and analysis procedures. These descriptions allow readers to judge the relevance of the findings to other EFL learning contexts.

### Treatment

The treatment phase which lasted for 14 90-min instructional sessions was designed to compare the effects of two instructional conditions on Chinese EFL learners’ vocabulary learning and academic enjoyment: augmented reality game-based instruction for the experimental group and traditional teacher-fronted instruction for the control group. In order to maintain comparability across conditions, both groups studied the same target vocabulary items and pursued the same instructional objectives. The main difference between the two groups lay in the mode of instruction and the nature of classroom engagement. Whereas the experimental group learned the target words through an AR-supported game-based application, the control group received conventional vocabulary instruction in a teacher-centered format, which reflects common instructional practice in many Chinese EFL classrooms. Thus, any differences observed between the two groups could be more plausibly attributed to the instructional treatment rather than to differences in content.

**Figure 1:**
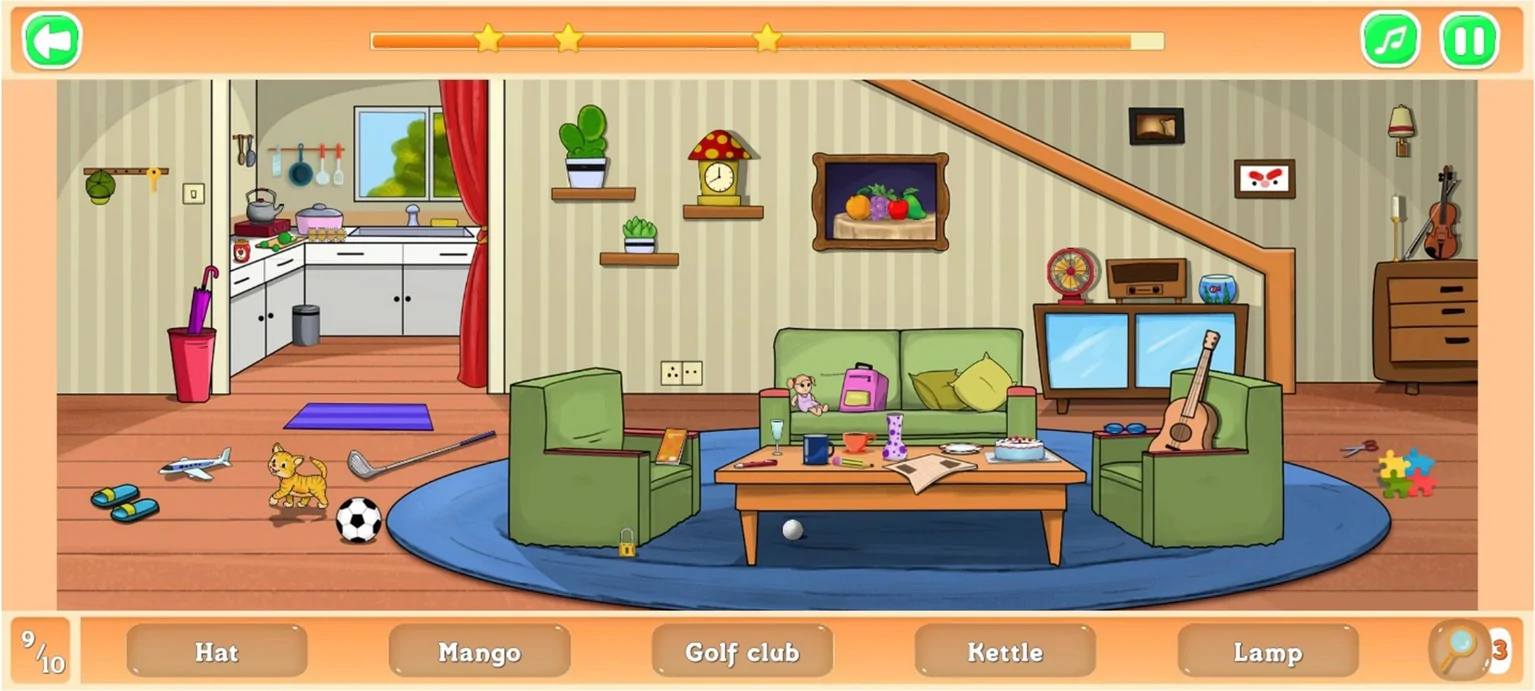
Built-in vocabulary recognition game in Vocabulary Builder AR.

**Figure 2:**
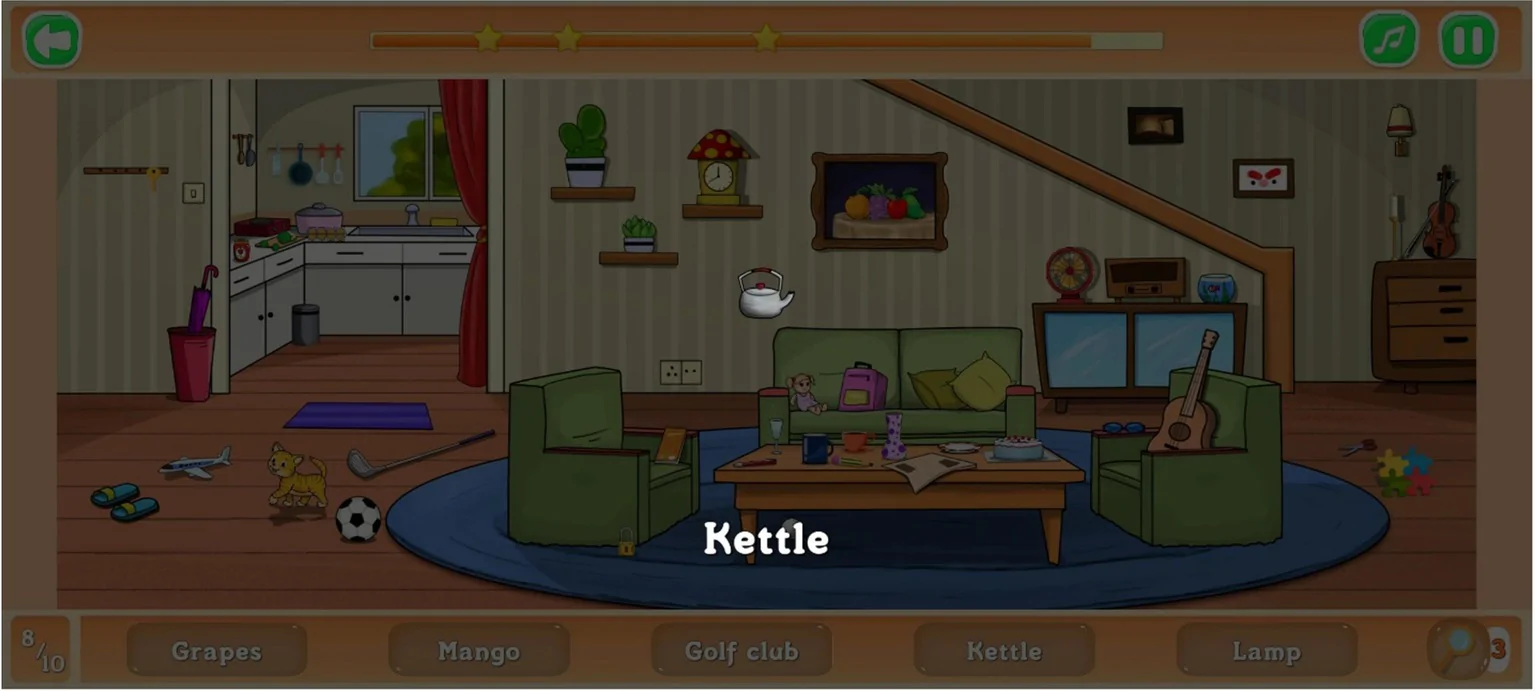
Immediate feedback after correct target-word selection in Vocabulary Builder AR.

**Figure 3:**
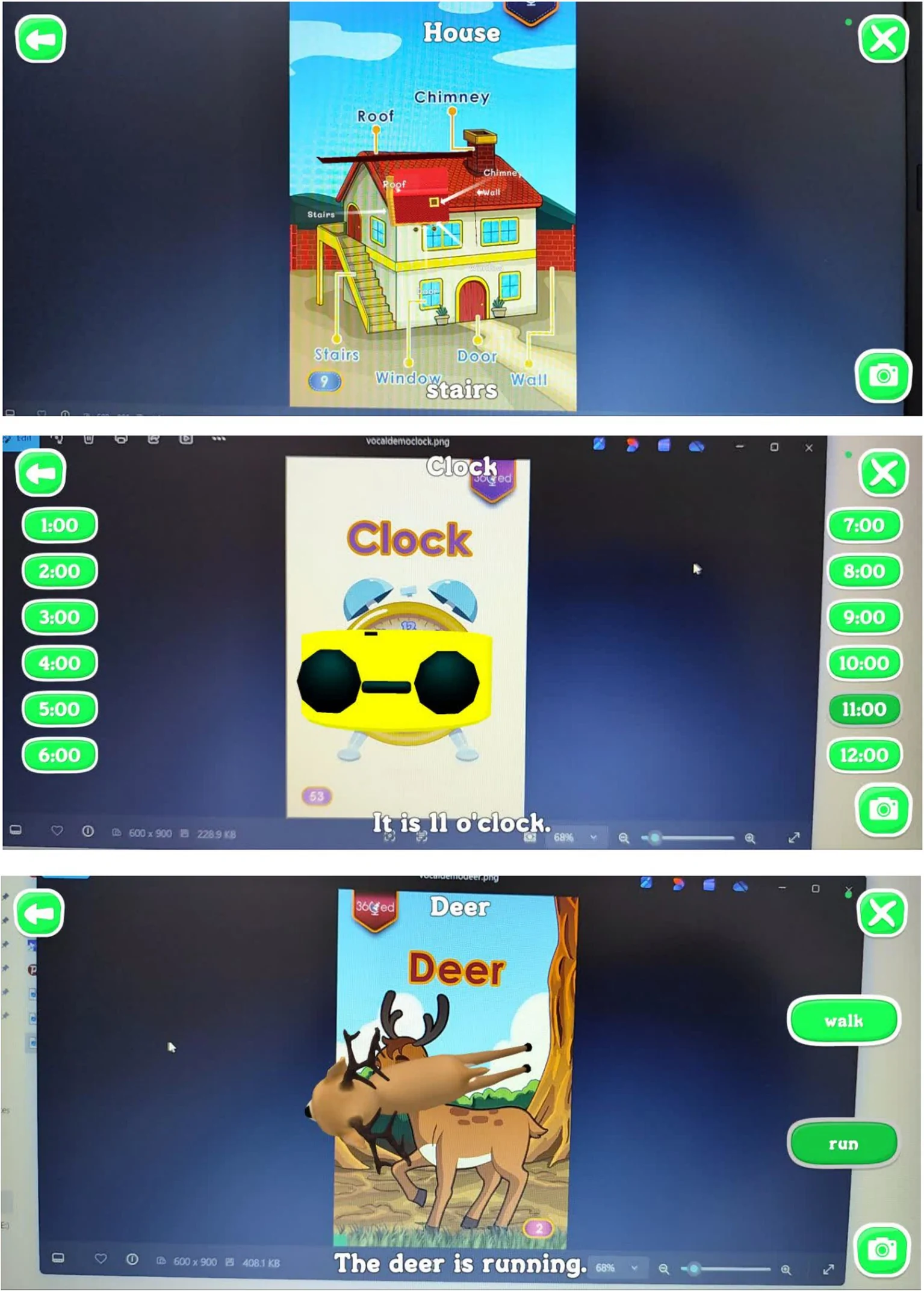
Examples of card-triggered three-dimensional vocabulary content in Vocabulary Builder AR. Figures 1–3 depict the standard, unmodified Vocabulary Builder AR interfaces and content used in the intervention. In the house example, learners positioned the device camera over the corresponding designated image card, after which the model appeared on the device screen. By selecting a component, such as the stairs, the relevant word was displayed and pronounced aloud, providing visual and auditory input. Similarly, the clock model allowed learners to select different times; the corresponding sentence then appeared and was pronounced. In the deer example, the model appeared after the application recognized the designated image card. Learners could select either the “walk” or “run” command, and the deer performed the corresponding movement. These models, commands, audio files, and interactions were built-in features of the application and were not created or modified by the researchers.

Before the intervention began, learners in the experimental group were introduced to Vocabulary Builder AR and shown how to install and use it on their Android or iOS devices. They were trained to open the application, position the device camera over the designated physical image cards, navigate the interface, listen to pronunciation, observe the preloaded three-dimensional representations, and complete the built-in practice tasks. The orientation concerned only the use of the existing learner-facing application; learners did not use any development software. Once they had become familiar with the application, the instructional sessions began. During the treatment period, the experimental group learned vocabulary through a sequence of activities that integrated visualization, pronunciation, contextualized exposure, interaction, and game-like feedback. For each target word, students presented the corresponding designated image card to the device camera. When the card image was recognized, the application displayed the associated preloaded three-dimensional model. This visual representation was accompanied by the pronunciation of the word and an example of its use in context. In this way, vocabulary learning moved beyond simple word-list memorization and involved multimodal exposure linking form, meaning, sound, and use. Figures 1–3 illustrate the built-in game interface and card-triggered content of Vocabulary Builder AR used in the intervention.

After this initial exposure, students engaged in interactive practice through the application. The built-in activities required learners to recognize, identify, and use the target vocabulary and provided immediate system-generated feedback. The application’s existing scoring feature tracked correct responses and introduced a playful and competitive element. The researchers used this feature as supplied and did not modify its rules or operation. Instructionally, the scoring system was used to sustain attention, encourage repeated engagement with the target words, and support self-monitoring and self-correction.

The experimental teacher’s role differed from that of a traditional instructor. Rather than serving primarily as the direct transmitter of vocabulary knowledge, the teacher acted as a facilitator of learning. At the beginning of each lesson, the teacher briefly introduced the instructional focus and clarified the goals of the session. During the AR activities, the teacher moved around the classroom, provided guidance when needed, answered technical or linguistic questions, and encouraged students to interact actively with the vocabulary tasks. The teacher also ensured that learners remained focused on the instructional objectives and used the application appropriately. In this sense, the AR game-based condition reflected a more learner-centered approach in which students constructed understanding through direct interaction with the learning materials, while the teacher provided scaffolding and support rather than constant direct explanation.

A typical lesson in the experimental group proceeded in several stages. First, the teacher introduced the lesson focus and prepared learners for the target vocabulary set. Second, students used Vocabulary Builder AR to present the corresponding designated image cards to the device camera and observe the preloaded three-dimensional models. Third, they listened to the pronunciation and attended to the contextualized usage examples provided in the application. Fourth, they completed the built-in interactive practice tasks designed to reinforce recognition and use of the target items. Fifth, they received the feedback and scores generated by the application, which encouraged further engagement with the material. Finally, the lesson concluded with a brief review in which students revisited problematic items and consolidated their learning. This recurring instructional sequence enabled learners to encounter each target word through visual, auditory, contextual, and interactive channels.

By contrast, the control group received traditional teacher-fronted vocabulary instruction, which served as the comparison condition. In this group, vocabulary teaching was delivered in the more conventional format typically associated with formal EFL instruction in China. The teacher introduced the target vocabulary items directly, explained their meanings, modeled their pronunciation, and provided example sentences. Students listened to the teacher’s explanations, repeated the words after the teacher, took notes, and completed textbook- or worksheet-based follow-up exercises. The learning process in this condition relied primarily on teacher explanation, verbal repetition, and written practice, rather than on technology-mediated interaction. Although control learners were exposed to the same target lexical items as experimental learners, they did not use Vocabulary Builder AR or its designated image cards, interact with three-dimensional objects, or receive application-generated game feedback and scores.

In the control condition, the teacher played the central instructional role throughout the lesson. Vocabulary presentation was mainly lecture-based and proceeded from explanation to controlled practice. After introducing the target items, the teacher typically clarified meanings through verbal definitions, translation equivalents, or example sentences and then asked students to repeat the words and record them in their notebooks. Practice activities were carried out in a traditional format, such as sentence completion, matching, teacher-led questioning, or other paper-based exercises. Feedback was also teacher-provided rather than system-generated. Compared with the experimental condition, therefore, the control group experienced a more passive learning environment in which the teacher managed the lesson flow and students had fewer opportunities for multimodal and exploratory engagement with the target vocabulary.

Despite the difference in instructional delivery, both groups were treated comparably in terms of curricular content and learning goals. The same vocabulary items were taught to both groups, and both conditions aimed at helping students understand word meanings, recognize correct pronunciation, and use vocabulary appropriately in context. However, the instructional experience differed substantially. In the AR game-based condition, vocabulary learning was embedded in an interactive, multisensory, and game-enhanced environment that encouraged exploration, repetition, and immediate feedback. In the traditional condition, vocabulary learning followed a teacher-led and form-focused pattern centered on explanation, repetition, and written reinforcement. This contrast constituted the core treatment difference examined in the present study.

From a methodological perspective, the treatment was designed not only to compare two teaching techniques but also to examine two different learning experiences. The experimental group was expected to engage with vocabulary in a more active, contextualized, and affectively supportive manner because the AR environment combined visual support, contextual information, interactivity, and game-like features. The control group, in contrast, represented the conventional instructional baseline against which the pedagogical value of AR game-based instruction could be evaluated. In this way, the treatment structure made it possible to investigate whether augmented reality game-based instruction could offer advantages over traditional teacher-fronted teaching with respect to learners’ vocabulary development and their academic enjoyment.

**Figure 4:**
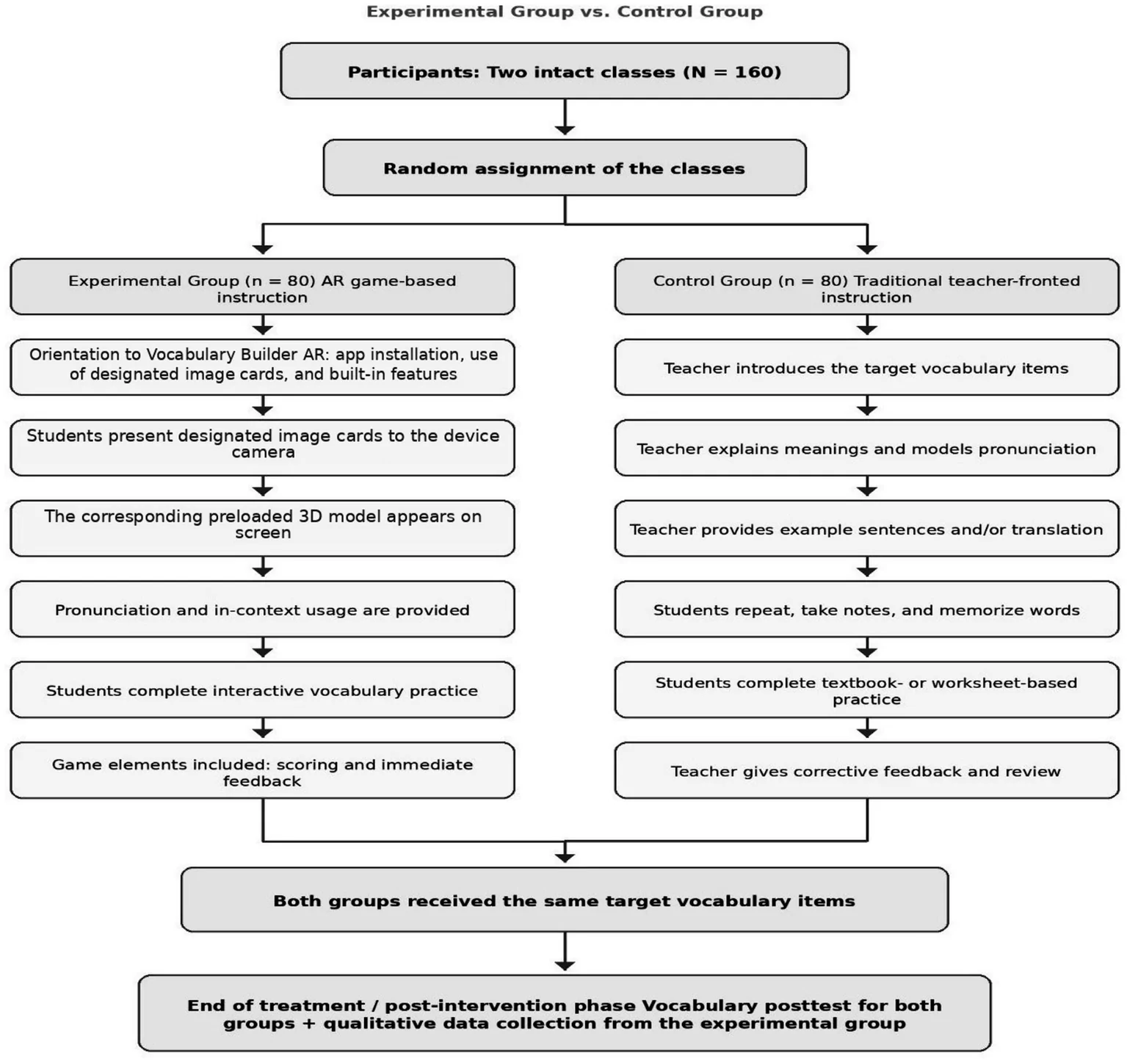
Flowchart of the treatment phase.

On the whole, several steps were taken to ensure consistency of implementation. Before the intervention, the instructional procedures for both groups were planned in detail, including the target vocabulary items, lesson sequence, practice activities, and assessment schedule. The teacher followed the same vocabulary in both groups, and the researchers monitored the instructional process to ensure that the experimental group used the AR game-based activities as intended and that the control group did not receive AR-supported instruction. Any technical problems observed during the AR sessions were addressed immediately so that they would not interfere with participation in the treatment. These procedures were used to maintain consistency across the 14-week intervention and to make the instructional conditions sufficiently clear for future replication. The treatment procedure is summarized in Figure 4.

### Data analysis procedures

The quantitative and qualitative data were analyzed in accordance with the concurrent mixed-methods design of the study. For the quantitative strand, a Kolmogorov–Smirnov test was first conducted to examine whether the vocabulary scores met the assumption of normality. The results indicated that the data were normally distributed; therefore, parametric statistics were considered appropriate. Since the study involved comparisons between two separate groups, independent-samples t-tests were employed for the quantitative analyses. An independent-samples t-test was first conducted on the pretest scores to determine whether the experimental and control groups were comparable prior to the treatment. A second independent-samples t-test was then performed on the posttest scores to compare the vocabulary performance of the two groups after the intervention and to determine whether augmented reality game-based instruction produced significantly different vocabulary learning outcomes from traditional teacher-fronted instruction.

For the qualitative strand, the narrative accounts and semi-structured interview data were analyzed thematically in order to explore how learners perceived and experienced academic enjoyment in the augmented reality game-based instructional context. The analysis was conducted through a systematic and recursive process. First, all narratives and interview transcripts were read several times in order to achieve close familiarity with the data and to develop an overall sense of recurring meanings, emotions, and patterns of experience. At this stage, the researchers paid particular attention to expressions related to enjoyment, interest, engagement, motivation, satisfaction, challenge, novelty, and emotional reactions to the AR-based activities. In the second stage, the data were coded line by line, and meaningful units of text were assigned initial labels that captured the essence of participants’ comments. These initial codes remained close to the participants’ own words and reflected repeated ideas across the dataset, such as increased interest in vocabulary learning, enjoyment of interaction with 3D objects, satisfaction with immediate feedback, reduced monotony compared with traditional instruction, and occasional difficulty or hesitation in using the technology.

In the next stage, the researchers examined the full set of codes and grouped conceptually related codes into broader categories. This step involved constant comparison across the narrative and interview data so that patterns emerging in one source could be checked against the other. Categories were not treated as final themes immediately; rather, they were reviewed repeatedly to determine whether they represented a coherent underlying idea relevant to the research question. Themes were achieved only when a cluster of related categories consistently captured a meaningful and recurring aspect of learners’ academic enjoyment across participants and across the two qualitative data sources. In other words, a theme was retained when it was supported by sufficient evidence in the data, showed internal coherence, was clearly distinguishable from other themes, and contributed directly to understanding how learners experienced enjoyment in the AR-supported instructional environment. After the candidate themes were identified, the researchers revisited the original narratives and interview excerpts to ensure that each theme was firmly grounded in the data and accurately represented the participants’ experiences. The themes were then refined, clearly defined, and named to reflect their substantive meaning in relation to academic enjoyment.

To enhance the trustworthiness of the qualitative analysis, the two researchers coded the narratives and interview transcripts independently before comparing their coding decisions. This independent coding process reduced the risk of subjective bias and increased the dependability of the interpretation. Inter-coder dependability was high, with a Cohen’s kappa coefficient of 0.85, indicating strong agreement between the coders. Disagreements were discussed until consensus was reached, and the final thematic structure was established only after this process of comparison, refinement, and confirmation. Through this procedure, the thematic analysis provided a rigorous account of the affective experiences reported by learners in the augmented reality game-based instructional setting.

## Results

### Impact of augmented reality game-based instruction to Chinese EFL learners’ vocabulary learning

To achieve the first objective of the study, the researchers attempted to measure how augmented reality game-based instruction enhances Chinese EFL learners’ vocabulary learning. To do so, before conducting the t-test to ensure the comparability of the learners’ baseline performance, a Kolmogorov–Smirnov Test was conducted to ensure the normality assumption – the results of which (see Table 1) showed no violation of the assumption on both time intervals (*p* > 0.05).

**Table 1:** One-sample Kolmogorov–Smirnov test.

		Baseline scores	Posttest scores
*N*		160	160
Normal parameters	Mean	6.556	8.382
	Std. deviation	2.155	3.420
Most extreme differences	Absolute	0.106	0.107
	Positive	0.106	0.107
	Negative	−0.100	−0.056
Kolmogorov–Smirnov Z		1.340	1.354
Asymp. sig. (2-tailed)		0.055	0.051

Table 2 reveals a broadly similar baseline pattern of performance between the experimental group (*M* = 6.287, *SD* = 2.208) and the control group (*M* = 6.825, *SD* = 2.080).

**Table 2:** Descriptive statistics of baseline vocabulary knowledge.

	Condition	*N*	Mean	Std. deviation	Std. error mean
Baseline scores	Experimental	80	6.287	2.208	0.246
	Control	80	6.825	2.080	0.232

Table 3 confirms the equality of variances through the Levene’s Test (*p* > 0.05). Furthermore, the table demonstrates an insignificant baseline difference between the two conditions (*t* = −1.584, *df* = 158, *p* > 0.05, *MD* = −0.537). For the baseline test, Cohen’s *d* = −0.25, which is a small effect. The negative sign simply means the control group’s mean was slightly higher than the experimental group’s mean before treatment. Since the corresponding *t*-test was nonsignificant, this supports the claim that the two groups were broadly comparable before the intervention.

**Table 3:** Independent samples test of baseline knowledge of vocabulary.

		Levene’s test for equality of variances		t-test for equality of means						
		F	Sig.	t	df	Sig. (2-tailed)	Mean difference	Std. error difference	95% confidence interval of the difference	
									Lower	Upper
Baseline scores	Equal variances assumed	0.402	0.527	−1.584	158	0.115	−0.537	0.339	−1.207	0.132
	Equal variances not assumed			−1.584	157.441	0.115	−0.537	0.339	−1.207	0.132

Table 4, however, indicates that the experimental group (*M* = 10.121, *SD* = 3.520) achieved higher lexical gains compared to the control group (*M* = 6.643, *SD* = 2.242) on the posttest.

**Table 4:** Descriptive statistics of vocabulary knowledge on the posttest.

	Condition	*N*	Mean	Std. deviation	Std. error mean
Posttest Scores	Experimental	80	10.121	3.520	0.393
	Control	80	6.643	2.242	0.250

Table 5 shows the violations of equality of variances confirmed through a Levene’s Test (*p* < 0.05). The table, nonetheless, confirms the statistical superiority of the experimental group over the control group (*t* = 7.453, *df* = 134.063, *p* < 0.05, *MD* = 3.478) following the augmented reality game-based treatment. For the posttest, Cohen’s *d* = 1.18, which is a large effect size. This means the experimental group’s posttest vocabulary performance was about 1.18 pooled standard deviations higher than the control group’s performance. That is a strong practical effect in favor of AR game-based instruction.

**Table 5:** Independent samples test of knowledge of vocabulary on the posttest.

		Levene’s test for equality of variances		t-test for equality of means						
		F	Sig.	t	df	Sig. (2-tailed)	Mean difference	Std. error difference	95% confidence interval of the difference	
									Lower	Upper
Posttest scores	Equal variances assumed	12.864	0.000	7.453	158	0.000	3.478	0.466	2.556	4.399
	Equal variances not assumed			7.453	134.063	0.000	3.478	0.466	2.555	4.401

### Chinese EFL learners’ perception and experience of academic enjoyment in augmented reality game-based instruction

The narrative accounts and semi-structured interview data were analyzed thematically to explore how Chinese EFL learners perceived and experienced academic enjoyment in the augmented reality game-based instructional context. The analysis provided three main themes: (1) perceived heightened interest through novelty and immersion, (2) perceived greater satisfaction through clearer understanding and vocabulary retention, and (3) perceived stronger motivation through interactivity and game-like engagement. Across the dataset, learners’ enjoyment was not described merely as fun or entertainment; rather, it emerged as a meaningful emotional response connected to curiosity, understanding, participation, and a growing sense of achievement in vocabulary learning.

#### Theme 1: Perceived heightened interest through novelty and immersion

The first theme showed that many learners experienced academic enjoyment because the AR-based instruction introduced a sense of novelty and immersion that was absent from their usual English vocabulary learning experiences. In their narratives, students repeatedly contrasted the AR lessons with traditional vocabulary instruction, which they often described as repetitive, teacher-dominated, and dependent on memorization. By contrast, the AR-supported learning environment appeared to transform vocabulary learning into a more vivid, stimulating, and engaging process.

One learner wrote in a narrative:

“In my previous English classes, vocabulary learning was usually a process of reading a word, checking its Chinese meaning, repeating it several times, and then trying to memorize it. I often felt that this process was mechanical and sometimes tiring, especially when there were many new words in one lesson. In the AR class, however, I felt something very different. When I scanned the card and suddenly saw the 3D object appear on my phone screen, I became curious immediately. It was not just a word in a book anymore. I felt that the word had become something real and visible. This made me want to continue the activity and find out more. I think this feeling of surprise and curiosity made the whole learning experience much more enjoyable for me.” (Narrative, Participant 12)

Another learner similarly reflected:

“What I enjoyed most was that the lesson felt alive. In a traditional class, the teacher explains the word, we write it down, and then we try to remember it. But in the AR activity, I could interact with the word in a new way. I could see it, hear it, and connect it to a situation. This made me feel more involved in the lesson. I was not just sitting and listening. I was exploring. Because of this, I did not feel bored or sleepy as I sometimes do in ordinary vocabulary classes. Instead, I felt interested from the beginning to the end of the activity.” (Narrative, Participant 27)

A third narrative excerpt highlighted the emotional dimension of this novelty:

“Usually, when I study vocabulary, I feel pressure because I know I have to memorize many words and I am afraid of forgetting them later. But during the AR lessons, I felt more relaxed and interested. The class atmosphere felt different because the activity was new and attractive. I think the technology itself made me pay more attention, but more importantly, it changed my mood in the class. I was more willing to participate and less afraid of making mistakes. This is why I can say that I really enjoyed learning in this way.” (Narrative, Participant 34)

The interview data reinforced these narrative accounts. One learner stated:

“I enjoyed the class because it did not feel like ordinary memorization. It felt more real and more exciting.” (Interview, Participant 8). Another participant explained: “The AR activities helped me stay focused because I wanted to see each vocabulary item and interact with it. That made the lesson more interesting than a normal class.” (Interview, Participant 19)

Taken together, these accounts suggest that novelty alone was not the only source of enjoyment. Rather, the immersive nature of the AR-supported environment helped learners become more attentive, emotionally engaged, and interested in vocabulary learning.

#### Theme 2: Perceived greater satisfaction through clearer understanding and vocabulary retention

The second theme revealed that learners’ academic enjoyment was strongly linked to the feeling that AR-supported instruction helped them understand vocabulary more clearly and remember it more effectively. In other words, enjoyment was tied not only to the attractive form of the instruction, but also to the learners’ perception that the instructional approach genuinely supported learning. Many participants described a sense of satisfaction when they realized that they could understand the target vocabulary more easily through the combination of 3D visualization, pronunciation support, and contextualized usage.

One participant wrote:

“For me, the most enjoyable part of the AR lessons was not only that they were interesting, but that they helped me understand the vocabulary in a better way. In many traditional classes, I may remember the translation of a word for a short time, but I do not always really understand how to use it. In the AR activity, I could see the object, listen to the pronunciation, and also notice how the word was used in context. These different kinds of information worked together and helped me build a clearer understanding. Because I understood the word more deeply, I felt more confident and satisfied. This feeling of understanding made the lesson enjoyable, because I felt that I was really learning something instead of only trying to memorize it.” (Narrative, Participant 5)

Another learner emphasized the role of comprehension in enjoyment:

“I found this method enjoyable because it reduced my confusion. Sometimes when I learn vocabulary from a list, I mix up words or forget them quickly. But when I saw the 3D image and heard the pronunciation, the meaning became more concrete in my mind. I could connect the word to an image and to an example, and this made it easier to remember later. I think I enjoyed the lesson more because I felt successful during the process. I was not guessing all the time. I could understand the words more clearly, and this made me feel happy and encouraged.” (Narrative, Participant 33)

A third learner wrote in a more reflective way:

“In my opinion, enjoyment in learning does not only come from having fun. It also comes from the feeling that the learning method is helping you progress. In the AR class, I often had the feeling that the vocabulary stayed in my mind more easily than before. Because the words were connected to images, sound, and practice, they seemed more memorable. This gave me a feeling of achievement. I enjoyed the lessons because they made me feel more capable as an English learner.” (Narrative, Participant 46)

The interview data showed a similar pattern. One interviewee commented:

“I enjoyed the AR activities because I understood the vocabulary more completely, not only the meaning but also the pronunciation and use.” (Interview, Participant 14). Another said: “When I could answer correctly after using the app, I felt satisfied. It was enjoyable because I could feel my learning progress.” (Interview, Participant 22)

These excerpts indicate that academic enjoyment was closely related to clarity, comprehension, and the perception of effective learning. Learners appeared to enjoy the AR environment partly because it helped reduce ambiguity and increased their sense of mastery over the target vocabulary.

#### Theme 3: Perceived stronger motivation through interactivity and game-like engagement

The third theme concerned the motivational force of the interactive and game-based features embedded in the AR instruction. Learners frequently reported that the scoring system, immediate feedback, and active participation made the learning process more dynamic and enjoyable. A key pattern in the narratives was that students appreciated being active participants rather than passive listeners. This active role appeared to make them feel more responsible for their learning and more emotionally invested in classroom tasks.

One learner wrote:

“A very important reason why I enjoyed the AR lessons was that I was not only receiving information from the teacher. I had to act, respond, check, and continue by myself. This made me feel that I was part of the learning process. The scoring system also added a sense of challenge. I wanted to do well, and when I got a good result, I felt excited. Even when I made a mistake, I wanted to try again because the activity encouraged me to continue. In normal classes, I often wait for the teacher’s explanation and do not feel very active. In this class, however, I felt more responsible for my own learning, and this made the experience more motivating and enjoyable.” (Narrative, Participant 9)

Another participant explained how interaction supported enjoyment:

“What made the class enjoyable for me was the feeling of doing something, not just listening. I needed to scan the image card, observe the object, listen carefully, and then complete the task. This process kept me engaged because every step required my attention. Also, the score gave me an immediate sense of whether I was doing well or not. I think this helped me stay motivated. It was almost like a game, but at the same time it was clearly related to learning. Because of this combination, I felt more energetic and involved than in a traditional lesson.” (Narrative, Participant 41)

A third narrative showed that challenge and enjoyment could coexist:

“At the beginning, I was a little slow in using the app, so I was not fully comfortable. However, after I became more familiar with it, I began to enjoy the lesson much more. I think one reason is that the activity gave me a challenge in a positive way. I wanted to complete the tasks correctly and improve my score. This was different from feeling pressure in the traditional sense. It was more like being encouraged to participate. Because I was active and interested, I felt more enjoyment during the lesson.” (Narrative, Participant 17)

Interview excerpts supported this theme as well. One learner remarked:

“The score made me want to continue. It gave me a reason to stay involved in the activity.” (Interview, Participant 11)

Another explained:

“In the AR class, I participated more than in normal classes. That made the lesson more enjoyable because I felt engaged all the time.” (Interview, Participant 30)

These findings suggest that academic enjoyment was enhanced by the game-like and interactive structure of the learning environment. Learners valued the sense of action, challenge, and immediate response, all of which contributed to stronger engagement and enjoyment.

Taken together, the quantitative and qualitative findings provide converging support for the value of augmented reality game-based instruction in the present study. Quantitatively, the results showed that although the experimental and control groups were comparable at the pretest stage, the learners in the experimental group significantly outperformed those in the control group on the posttest, indicating that AR game-based instruction had a positive effect on Chinese EFL learners’ vocabulary learning. Qualitatively, the thematic analysis further revealed that learners experienced academic enjoyment through three interrelated perceived dimensions: heightened interest through novelty and immersion, higher satisfaction through clearer understanding and vocabulary retention, and stronger motivation through interactivity and game-like engagement. Overall, these findings suggest that augmented reality game-based instruction was beneficial not only in supporting vocabulary development but also in fostering positive emotional experiences that made the learning process more engaging, meaningful, and enjoyable for Chinese EFL learners.

## Discussion

The present study was designed to address two closely related objectives: first, to examine whether augmented reality game-based instruction affects Chinese EFL learners’ vocabulary learning, and second, to explore how Chinese EFL learners perceive and experience academic enjoyment in such an instructional environment. Taken together, the findings indicate that the AR game-based condition was associated with superior vocabulary performance and with a cluster of positive affective experiences, namely perceived heightened interest through novelty and immersion, perceived enhanced satisfaction through clearer understanding and vocabulary gain, and perceived increased motivation through interactivity and game-like engagement. Viewed as a whole, these results support the argument advanced in some of the reviewed studies that augmented reality game-based instruction can contribute to both the cognitive and emotional dimensions of language learning, especially when it is implemented in a pedagogically meaningful manner.

The findings can be interpreted in direct relation to the theoretical foundations of the study. In response to the first research question, the quantitative results showed that learners in the AR game-based condition outperformed those in the traditional teacher-fronted condition on the vocabulary posttest. This finding supports the constructivist assumption that vocabulary learning can be strengthened when learners engage actively with multimodal and contextualized input rather than receive vocabulary knowledge passively. The AR-supported activities required learners to use designated image cards with the application, observe preloaded three-dimensional representations, listen to pronunciation, attend to contextualized examples, complete built-in interactive tasks, and receive immediate feedback. These processes reflect active knowledge construction and help explain why the experimental group achieved stronger vocabulary outcomes. In response to the second research question, the qualitative findings showed that learners experienced academic enjoyment through novelty and immersion, clearer understanding and retention, and interactivity and game-like engagement. These findings support the Affective Filter Hypothesis by suggesting that the AR game-based environment created more positive emotional conditions for learning. Learners’ increased interest, satisfaction, confidence, and motivation may have lowered affective barriers and made them more willing to engage with vocabulary input. Thus, the findings show that the cognitive and affective outcomes of the study are theoretically connected rather than separate.

The first major finding was that the experimental group significantly outperformed the control group on the vocabulary posttest. This result suggests that augmented reality game-based instruction was more effective than traditional teacher-fronted instruction in supporting vocabulary learning in the present Chinese EFL context. This finding is broadly consistent with previous studies that have reported favorable effects of AR-supported language learning. Most directly, it aligns with Hung and Yeh (2023), who found that learners receiving AR game-supported instruction performed better than those receiving paper-and-pen-based instruction on vocabulary knowledge. It is also in line with Jia et al. (2025), whose findings showed that AR-supported game-based learning was particularly beneficial for academic English vocabulary learning and long-term retention. Likewise, the present result is compatible with Khazaie and Ebadi (2025), who reported improved comprehension in an AR-supported gamified flipped learning context, and with Namaziandost and Çakmak (2026), who found that an AR game-supported flipped learning model improved vocabulary retention and oral fluency while reducing cognitive burden. At a broader level, the current quantitative result also accords with earlier work indicating that AR can positively influence academic performance and English learning (Lee, 2012; Ndrurua et al., 2025). In this respect, the present study extends the still limited evidence base concerning AR game-based vocabulary instruction in Chinese EFL settings and offers support for the view that such instruction can promote measurable lexical development.

At the same time, the present vocabulary finding should be interpreted in relation to the more mixed conclusions reported in the literature. In particular, Raghav Bhat et al. (2025) argued that although AR can enhance engagement through enriched input and attractive design, such engagement does not necessarily translate into stronger learning outcomes. The current findings differ from that pattern, but they do not contradict it. Rather, they reinforce the argument that the effectiveness of AR is not an inherent property of the technology itself, but depends on how purposefully it is integrated into instruction. In the present study, the AR treatment was not limited to visually appealing presentation. It combined 3D modeling, pronunciation, in-context usage, interactive practice, and a scoring system in a structured instructional sequence. This design likely enabled learners to process vocabulary through multiple channels and to encounter words in a more meaningful and contextualized manner than was possible in the teacher-fronted condition. Accordingly, the present results lend support to the claim that AR becomes pedagogically effective when it is deliberately designed to scaffold learning, rather than when it is used as a technological add-on (Raghav Bhat et al., 2025).

This quantitative finding can also be explained in light of constructivism, which formed one of the theoretical foundations of the study. From a constructivist perspective, learning is most effective when learners actively construct knowledge through interaction, contextualization, and meaning-making rather than passively receiving information. The AR game-based environment used in the present study appears to have supported exactly these processes. By using the designated image cards with the application, observing preloaded three-dimensional representations, listening to pronunciation, attending to contextualized usage, and completing built-in practice tasks, learners were encouraged to build associations among form, meaning, sound, and use. This kind of engagement is consistent with the argument that learning is shaped by the relationships among learners, environments, objects, and actions (Dunleavy and Dede, 2014). It also aligns with Robinson and Coltz’s (2013) view that AR can promote constructivist learning by enabling direct engagement with interactive materials, with Wang et al.’s (2018) suggestion that AR helps learners integrate unfamiliar concepts into existing knowledge structures, and with Belda-Medina’s (2022) emphasis on learner-centered and experiential learning through AR. From this perspective, the stronger vocabulary performance of the experimental group can be understood not simply as the outcome of exposure to technology, but as the result of more active, situated, and multimodal knowledge construction.

The small decrease observed in the control group’s posttest mean also deserves careful comment. Although the control group’s mean score was slightly lower on the posttest than on the pretest, this pattern should not be taken to mean that traditional teacher-fronted instruction caused a decline in vocabulary learning. The decrease was small, and in the absence of a within-group inferential analysis, it should be interpreted cautiously. A more plausible explanation is that this slight fluctuation may partly reflect a chance factor associated with the structure of the vocabulary test itself. Because the instrument included matching and multiple-choice items, some responses may have been affected by guessing, which could produce minor score variation across administrations. In this sense, the slight reduction in the control group’s mean should not be overinterpreted as evidence that traditional instruction was harmful; rather, the more defensible conclusion is that the AR game-based group achieved a clearly stronger level of posttest performance relative to the comparison group.

The qualitative findings add an important affective layer to the interpretation of the study. The first theme, perceived heightened interest through novelty and immersion, indicates that learners experienced AR game-based instruction as more vivid, stimulating, and engaging than conventional vocabulary teaching. This finding is highly compatible with earlier research suggesting that AR can positively influence learners’ motivation and attitudes (Norouzifard et al., 2022) and can enhance engagement by providing enriched, context-sensitive, and interactive learning experiences (Saleh et al., 2025; Sun et al., 2024; Chen et al., 2025). It also converges with Amorim et al. (2022), whose review showed that AR is associated with engagement and positive affective outcomes, and with Ou Yang et al. (2023), who found greater enjoyment of learning in an AR-based condition. The present participants’ accounts suggest that academic enjoyment began with curiosity and surprise but developed into sustained involvement because the AR environment made vocabulary items feel more concrete and alive. This helps explain why learners repeatedly contrasted the AR condition with more routine, memorization-oriented classroom experiences. In other words, novelty may have captured attention initially, but immersion appears to have sustained learners’ interest over time. This interpretation is also consistent with the broader argument that games can make the teaching-learning process more engaging, interactive, and immersive (Hung et al., 2018).

The second theme, perceived enhanced satisfaction through clearer understanding and vocabulary retention, further strengthens the study’s contribution by showing that learners’ enjoyment was tied not only to fun, but also to comprehension and perceived learning success. This is important because it suggests that enjoyment in the present study was pedagogically meaningful rather than superficial. Learners appeared to feel satisfied when the AR environment helped them understand vocabulary more clearly and remember it more effectively. This finding fits well with the conceptualization of academic enjoyment as students’ positive emotional response to learning activities and educational experiences (Hascher and Hagenauer, 2011; Lumby, 2011; Ömeroğulları and Gläser-Zikuda, 2021). It also aligns with Pekrun’s (2006) view that enjoyment is associated with the successful accomplishment of tasks and with Elahi Shirvan et al.’s (2020) argument that enjoyment is a multifaceted construct involving cognitive, motivational, and psychological dimensions. In the present study, learners’ narratives suggest that the combination of visual, auditory, and contextual support reduced confusion and increased confidence, which in turn generated a stronger feeling of satisfaction. This interpretation is consistent with Amorim et al. (2022), who linked AR to long-term vocabulary retention and feelings of success, and with Jia et al. (2025), who reported positive effects of AR-supported game-based learning on vocabulary retention. Thus, the qualitative data help explain why the experimental group may have benefited cognitively: learners were not only exposed to vocabulary in richer ways, but also experienced the process of understanding as more rewarding.

The third theme, perceived increased motivation through interactivity and game-like engagement, likewise resonates strongly with the literature reviewed in the Introduction. A central insight from the qualitative data was that learners valued being active participants rather than passive recipients of teacher explanation. The scoring system, immediate feedback, and interactive tasks appear to have created a sense of challenge, involvement, and personal investment, all of which enhanced enjoyment. This finding is in line with work on digital game-based language learning showing that game-supported environments can foster motivation, engagement, and other positive learning outcomes (Hung et al., 2018; Xiong et al., 2024; Zou et al., 2021). It also echoes the literature on mobile game-based language learning, which emphasizes the educational promise of combining the accessibility of mobile devices with the immersive and engaging qualities of games (Chen et al., 2019; Elaish et al., 2019; Godwin-Jones, 2014; Wu, 2021). Since the AR treatment in the present study was implemented through mobile devices and included game-like scoring and immediate response features, it is reasonable that learners experienced higher motivation and more active involvement. This is also compatible with empirical work showing that mobile games can support vocabulary and other language skills (Amorim et al., 2022; Kazu and Kuvvetli, 2024; Li, 2021; Roohani and Heidari Vincheh, 2021; van Uittert et al., 2022). The present study therefore contributes to this line of research by showing that when mobile AR is combined with game-based design, learners may perceive the classroom not only as more engaging, but also as more motivating and participatory.

These affective findings can also be meaningfully interpreted through Krashen’s (1982) Affective Filter Hypothesis. According to this perspective, learners are more likely to process and acquire language input when they experience positive emotions such as enjoyment, confidence, and motivation, because such states lower the affective filter. The qualitative findings of the present study strongly suggest that the AR game-based environment fostered these favorable emotional conditions. Learners reported higher interest, greater satisfaction, and stronger motivation, all of which point to a classroom atmosphere that was more enjoyable and less monotonous than traditional vocabulary instruction. This interpretation is fully compatible with the view that positive emotions facilitate language learning by making learners more open to input (Krashen, 1982). It also aligns with Dewaele and MacIntyre’s (2016) conceptualization of enjoyment as a positive emotional force in language learning and with Jiang and Dewaele (2019), who highlighted the importance of such emotions for learners’ engagement and well-being. Moreover, the present findings support Dewaele et al.’s (2018) argument that enjoyment is dynamic and shaped by the learning environment, as well as Khajavy et al.’s (2018) observation that learners with higher enjoyment tend to participate more actively. In the present study, participation, engagement, and enjoyment appear to have reinforced one another, suggesting that the affective benefits of AR game-based instruction may have helped create better conditions for vocabulary learning.

Another point worth emphasizing is that the quantitative and qualitative findings do not stand as isolated outcomes; rather, they appear to complement and explain one another. The superior vocabulary performance of the experimental group can be better understood in light of learners’ reported interest, satisfaction, and motivation, just as the reported enjoyment becomes more meaningful when viewed alongside measurable vocabulary gains. This interplay is important because it reflects the dual theoretical grounding of the study. Constructivism helps explain how AR game-based instruction may support deeper learning through active, contextualized, and learner-centered engagement, while the Affective Filter Hypothesis helps explain why an enjoyable and motivating environment may make learners more receptive to language input. The current findings suggest that these two processes likely worked together. Learners may have performed better because they were cognitively and affectively engaged at the same time, and they may have enjoyed the instruction more because they felt they were learning successfully. This reciprocal relationship also reflects the broader positive psychology view that enjoyment is closely intertwined with accomplishment, motivation, and educational well-being (Elahi Shirvan et al., 2020; Pekrun, 2006).

A critical interpretation of these findings suggests that the positive effect of AR game-based instruction should not be attributed to the technology itself, but to the way the technology reorganized the vocabulary-learning process. In the experimental condition, learners encountered vocabulary through visual representation, pronunciation support, contextualized examples, interactive practice, immediate feedback, and game-like scoring. These features may have promoted deeper lexical processing by helping learners connect word form, meaning, sound, and use through multiple channels. Therefore, the findings should be understood not as evidence that AR automatically improves vocabulary learning, but as evidence that AR can become pedagogically effective when it is embedded in a structured instructional sequence aligned with clear language-learning objectives.

The qualitative findings also require interpretation beyond the general claim that learners enjoyed the AR-supported lessons. Although novelty and immersion initially appeared to attract learners’ attention, their enjoyment seemed to become educationally meaningful when they perceived that the AR activities helped them understand, remember, and use vocabulary more effectively. In this sense, academic enjoyment was closely connected to perceived learning success, participation, feedback, and a sense of achievement. At the same time, these findings should be interpreted cautiously. The study does not suggest that AR game-based instruction is universally effective in all contexts; rather, its effectiveness may depend on the quality of instructional design, teacher scaffolding, learner readiness, and the alignment between game elements and vocabulary-learning goals.

Beyond confirming previous findings on the value of AR and game-based learning, the present study makes several theoretical and pedagogical contributions. Theoretically, the study contributes to technology-enhanced language learning by showing how the cognitive and affective dimensions of vocabulary learning can operate together in an AR game-based environment. Rather than treating vocabulary achievement and academic enjoyment as separate outcomes, the findings suggest that they may be mutually reinforcing. Learners’ qualitative accounts showed that enjoyment was not limited to technological novelty or entertainment; instead, it was closely connected to clearer understanding, perceived retention, active participation, immediate feedback, and a sense of learning progress. This finding extends existing discussions of academic enjoyment in SLA by showing that enjoyment in digitally mediated environments can emerge from learners’ perception that the instructional design genuinely supports comprehension and achievement.

The study also contributes theoretically by bringing constructivism and the Affective Filter Hypothesis into closer dialogue. From a constructivist perspective, the AR game-based environment supported vocabulary learning by allowing learners to interact actively with multimodal input, connect words with visual and auditory representations, and construct meaning through contextualized practice. From the perspective of the Affective Filter Hypothesis, the same environment appeared to create more favorable emotional conditions by increasing interest, satisfaction, confidence, and motivation. The findings therefore suggest that AR game-based instruction may be effective not simply because it provides richer technological input, but because it can simultaneously promote active knowledge construction and lower affective barriers to language learning. In this sense, the study offers a more integrated explanation of how AR game-based instruction may support EFL vocabulary learning.

Pedagogically, the findings indicate that AR should not be introduced into language classrooms merely as a novel technological tool. Its instructional value depends on purposeful design. In the present study, the pedagogical strength of the AR treatment lay in the combination of 3D visualization, pronunciation support, contextualized usage, interactive vocabulary practice, scoring, immediate feedback, and teacher facilitation. These features worked together to transform vocabulary learning from a mainly memorization-based activity into a more interactive, multisensory, and emotionally engaging experience. Therefore, one practical contribution of the study is that it identifies design principles that may guide teachers and material developers when integrating AR into EFL vocabulary instruction.

More specifically, the findings suggest that effective AR game-based vocabulary instruction should include repeated exposure to target words, meaningful visual and auditory support, opportunities for learner interaction, immediate feedback, and teacher scaffolding. The teacher’s role remains important because learners need guidance to connect the AR activities with language-learning objectives. Thus, the study challenges a purely technology-driven view of AR implementation and instead supports a pedagogy-driven approach in which AR, game elements, and teacher support are aligned with clear vocabulary-learning goals. This contribution is particularly relevant for Chinese EFL contexts, where vocabulary learning is often associated with memorization, repetition, and teacher-fronted explanation. The present findings suggest that AR game-based instruction can provide a complementary approach that supports vocabulary development while also fostering academic enjoyment.

Overall, the present study extends previous scholarship by providing context-sensitive evidence from a Chinese EFL setting in which both vocabulary learning and academic enjoyment were examined together. This is particularly important given Xu et al.’s (2020) observation that much of the research on digital game-based language learning has been concentrated in the United States and Taiwan, leaving other contexts less fully explored. The current findings therefore contribute to filling that gap. At the same time, the study reinforces a point already emphasized in the literature: AR is not automatically beneficial. Its value lies in its instructional design. The success of the present intervention appears to stem from the fact that the AR environment was interactive, contextualized, multimodal, and game-enhanced, rather than from the mere presence of technology. In this sense, the study supports the broader view that AR game-based instruction can be educationally effective when it is aligned with sound pedagogical principles and when it promotes both meaningful language engagement and positive emotional experience.

## Conclusion and implications

The study examined whether augmented reality game-based instruction affects Chinese EFL learners’ vocabulary learning and explored how learners perceive and experience academic enjoyment in such an instructional environment. The findings showed that the learners who received AR game-based instruction outperformed those who experienced traditional teacher-fronted instruction on the vocabulary posttest, while the qualitative results further revealed that the AR-supported learning environment was associated with perceived heightened interest through novelty and immersion, perceived enhanced satisfaction through clearer understanding and vocabulary gain, and perceived increased motivation through interactivity and game-like engagement. Taken together, these findings suggest that augmented reality game-based instruction can be a valuable pedagogical approach for supporting both the cognitive and affective dimensions of English language learning. More specifically, the study indicates that when AR is integrated into instruction through purposeful game-based design, it can move beyond technological novelty and function as a meaningful instructional tool that promotes vocabulary development and fosters enjoyable learning experiences.

The findings carry several practical implications for educational stakeholders. For teachers, the results suggest that AR game-based instruction can serve as an effective supplement to conventional vocabulary teaching by making lexical learning more interactive, contextualized, and motivating; accordingly, teachers may consider integrating AR-supported tasks that combine visualization, pronunciation, contextual usage, and feedback into their classroom practices. For curriculum developers, the findings highlight the value of designing language-learning materials that incorporate multimodal and game-based features in pedagogically coherent ways rather than treating technology as an optional add-on. For policymakers and educational decision-makers, the study points to the need for greater institutional support for the integration of emerging technologies in EFL education, including the provision of digital infrastructure, access to suitable mobile platforms, and professional development opportunities that enable teachers to use AR meaningfully and confidently. More broadly, the study suggests that technology-enhanced language programs may be most effective when they are designed not only to improve achievement but also to promote positive emotional engagement, which is central to sustained participation and successful learning.

Notwithstanding its contributions, this study has several limitations that should be acknowledged. First, the study was conducted at a single university in China and involved two intact EFL classes. Although this design was appropriate for preserving the natural classroom context, it limits the generalizability of the findings to other institutions, age groups, proficiency levels, and cultural settings. In addition, randomization was conducted at the class level rather than at the individual level, which means that unmeasured class-level differences may have influenced the results despite the baseline comparability of the two groups. Second, the intervention lasted 14 weeks and examined vocabulary learning through immediate posttest performance. Therefore, the study cannot determine whether the observed vocabulary gains would be maintained over a longer period. Future research should include delayed posttests to examine long-term vocabulary retention and should consider longitudinal designs to explore whether learners’ academic enjoyment remains stable after the initial novelty of AR use decreases.

Third, the study focused specifically on vocabulary learning and academic enjoyment. Other relevant cognitive, affective, and behavioral variables, such as anxiety, self-confidence, willingness to communicate, learner autonomy, technology acceptance, cognitive load, and self-directed learning, were not directly measured. Although motivation, technology adoption, and self-directed learning were discussed in the conceptual framework, they were not operationalized as separate quantitative variables in the present study. Future studies could use validated scales, learning analytics, classroom observations, or structural models to examine how these constructs interact in AR-supported language learning environments. Fourth, the qualitative findings were based on learner narratives and semi-structured interviews, which provided valuable insight into participants’ experiences but were still dependent on self-report. Learners’ responses may have been influenced by social desirability, memory, or positive attitudes toward novel technology. Future research could strengthen the evidence by combining self-report data with classroom observation, screen-recorded interaction data, teacher logs, or behavioral indicators of engagement.

Finally, the findings should not be interpreted as evidence that AR game-based instruction is automatically superior to traditional instruction in all contexts. The positive results of the present study may have depended on the specific design of the intervention, including 3D visualization, pronunciation support, contextualized examples, interactive practice, immediate feedback, scoring, and teacher facilitation. Different AR tools, different vocabulary materials, limited technological infrastructure, or insufficient teacher support may produce different outcomes. Future research should therefore compare different forms of AR-supported pedagogy, examine treatment fidelity more systematically, and investigate which design features are most important for supporting vocabulary learning and academic enjoyment. These limitations suggest that the present findings should be viewed as promising but context-bound evidence that requires further replication and extension.

## Data Availability

The raw data supporting the conclusions of this article will be made available by the authors, without undue reservation.
